# Effects of Revision Rod Position on Spinal Construct Stability in Lumbar Revision Surgery: A Finite Element Study

**DOI:** 10.3389/fbioe.2021.799727

**Published:** 2022-01-05

**Authors:** Quan-Chang Tan, Jin-Feng Huang, Hao Bai, Zi-Xuan Liu, Xin-Yi Huang, Xiong Zhao, Zhao Yang, Cheng-Fei Du, Wei Lei, Zi-Xiang Wu

**Affiliations:** ^1^ Department of Orthopaedics, Xijing Hospital, The Air Force Medical University, Xi’an, China; ^2^ Department of Orthopaedics, Air Force Hospital of Eastern Theater Command, Nanjing, China; ^3^ Tianjin Key Laboratory for Advanced Mechatronic System Design and Intelligent Control, School of Mechanical Engineering, Tianjin University of Technology, Tianjin, China; ^4^ National Demonstration Center for Experimental Mechanical and Electrical Engineering Education, Tianjin University of Technology, Tianjin, China

**Keywords:** biomechanics, finite element analysis, construct configuration, cortical bone trajectory, dual-rod, satellite rod

## Abstract

Revision surgery (RS) is a necessary surgical intervention in clinical practice to treat spinal instrumentation–related symptomatic complications. Three constructs with different configurations have been applied in RS. One distinguishing characteristic of these configurations is that the revision rods connecting previous segments and revision segments are placed alongside, outside, or inside the previous rods at the level of facetectomy. Whether the position of the revision rod could generate mechanical disparities in revision constructs is unknown. The objective of this study was to assess the influence of the revision rod position on the construct after RS. A validated spinal finite element (FE) model was developed to simulate RS after previous instrumented fusion using a modified dual-rod construct (DRCm), satellite-rod construct (SRC), and cortical bone trajectory construct (CBTC). Thereafter, maximum von Mises stress (VMS) on the annulus fibrosus and cages and the ligament force of the interspinous ligament, supraspinous ligament, and ligamentum flavum under a pure moment load and a follower load in six directions were applied to assess the influence of the revision rod position on the revision construct. An approximately identical overall reducing tendency of VMS was observed among the three constructs. The changing tendency of the maximum VMS on the cages placed at L4-L5 was nearly equal among the three constructs. However, the changing tendency of the maximum VMS on the cage placed at L2-L3 was notable, especially in the CBTC under right bending and left axial rotation. The overall changing tendency of the ligament force in the DRCm, SRC, and CBTC was also approximately equal, while the ligament force of the CBTC was found to be significantly greater than that of the DRCm and SRC at L1-L2. The results indicated that the stiffness associated with the CBTC might be lower than that associated with the DRCm and SRC in RS. The results of the present study indicated that the DRCm, SRC, and CBTC could provide sufficient stabilization in RS. The CBTC was a less rigid construct. Rather than the revision rod position, the method of constructing spinal instrumentation played a role in influencing the biomechanics of revision.

## Introduction

Posterior instrumented fusion has been commonly accepted as treatment for spinal disorders due to degeneration, tumors, fractures, and deformities (Kaiser et al., 2014). Although the application of instrumented fusion has yielded positive clinical outcomes in treating spinal disorders, complications such as adjacent segment disease (ASD), proximal junctional kyphosis (PJK), and implant failure continue to be main concerns in clinical practice (Shinohara et al., 2016; Nicholls et al., 2017; Yamato et al., 2018; Hashimoto et al., 2019). With the increasing life expectancy of patients, an increasing number of revision surgeries (RS) is needed to relieve complication-related symptoms or to rescue primary implantations to maintain spinal stability (Rodriguez et al., 2014; Nicholls et al., 2017; Yamato et al., 2018). It has been reported that the rate of RS increased with time from 7.4% at the 1-year follow-up to 22.6% at the 4-year follow-up (Cecchinato et al., 2020).

**FIGURE 1:**
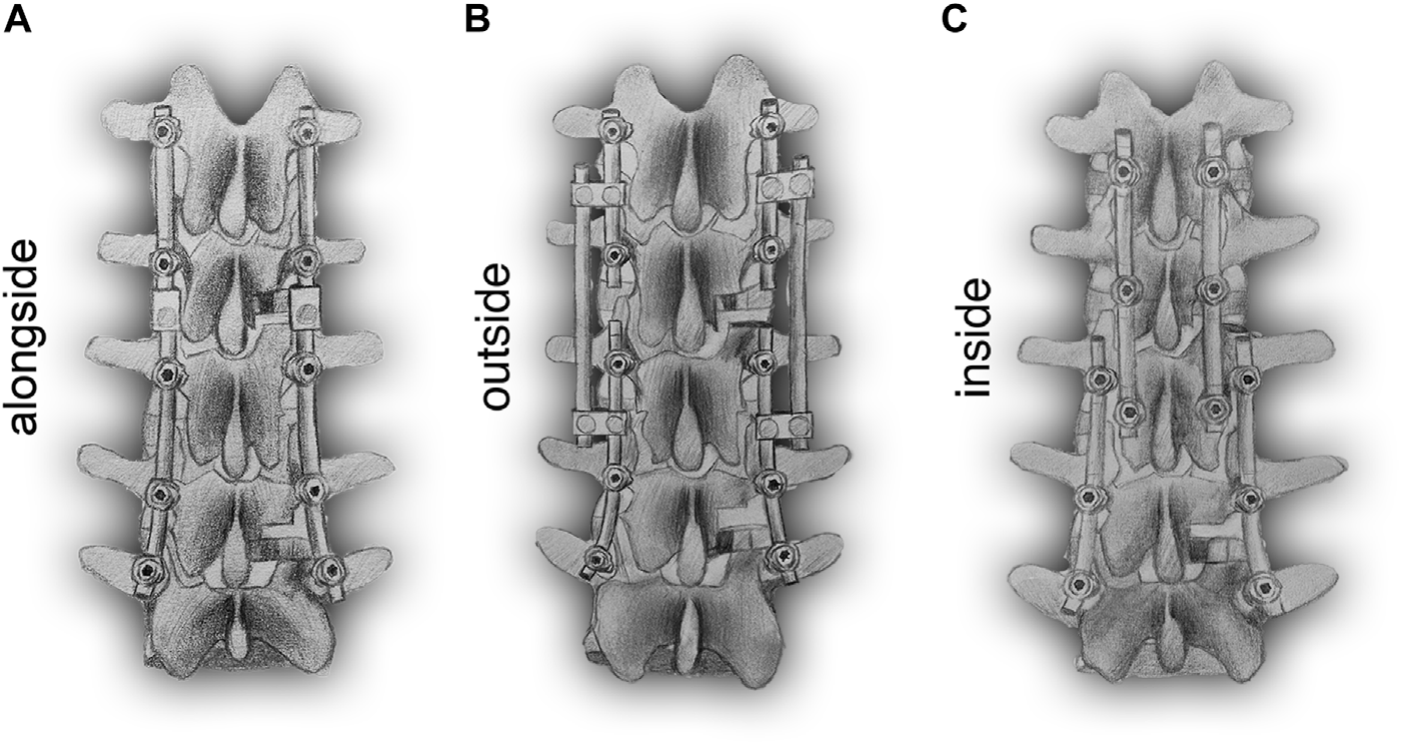
Schematic posterior-anterior illustration of revision models with revision rods placed alongside **(DRCm, A)**, outside **(SRC, B)**, and inside **(CBTC, C)** the previous rods.

Various constructs have been adopted in RS, including the conventional dual-rod construct (DRC) and its modified configuration (DRCm), satellite-rod construct (SRC), and cortical bone trajectory construct (CBTC). The DRC is the most common and standard conventional method to perform RS by replacing the prior rods with new longer rods, which could inevitably result in longer surgical duration, more blood loss, and higher risk of postoperative complications (Tan et al., 2021b). Retaining previous implants might reduce the risk of the aforementioned problems in that the integrity of the primary surgical site would be preserved. In addition, facetectomy was reported to be correlated with spinal stability (Ahuja et al., 2020), and whether the deferent position of placing the revision rod at the level of facetectomy could affect fixated spinal stability is still unknown. The DRCm is a modified dual-rod configuration that extends spinal fusion and instrumentation by connecting the revision rod to the previous rod alongside of it at the level of facetectomy where the site of the primary surgery (PS) and RS are connected. The SRC connects the revision rod to the previous rod using side-to-side connectors and affixed rods on the outside of it. The CBTC is a posterior instrumented technique that achieves spinal fixation in a novel way of placing screws in a medial-to-lateral orientation (cortical bone trajectory, CBT) with the screw’s tail closer to the spinous process; therefore, the revision rod is located inside of the primary rod (Figure 1). However, there is limited knowledge about the influence of different revision rod positions on the mechanical properties of spinal constructs after RS. Therefore, the aim of our study was to perform an FE analysis to compare the biomechanics of the DRCm, SRC, and CBTC in RS and assess the influence of the revision rod position on spinal stability after surgery, which could provide a basis for surgical type choice.

## Methods and Materials

### Generation and Validation of the Intact FE Model

A previously validated intact T12-L5 FE model was used in the present study (Tan et al., 2021a). Detailed modeling procedures are described briefly as follows: the geometrically intact thoracolumbar model was constructed in Mimics 10.0 (Materialise Technologies, Leuven, Belgium) from computed tomography images of a healthy 30-year-old healthy male subject without spinal abnormalities. Geometric model reconstruction was performed using the reverse engineering software Geomagic Studio 10.0 (Geomagic Inc., NC, United States). The creation of tetrahedral and hexahedral elements on the vertebrae and the assignment of material properties were completed using preprocessing software Hypermesh11.0 (Altair Engineering Corp., MI, United States). Subsequently, the FE model was created in Abaqus 6.11 (Dassault Systems Corp., PA, United States) for further analysis. The intact FE model was composed of the cancellous bone, cortical bone, the endplate, intervertebral disc, and posterior elements. The cancellous bone of the vertebra was surrounded by one layer of 0.35 mm cortical bone. The intervertebral disc was composed of nucleus pulposus and annulus fibrosus with reported proportions of 44 and 56%, respectively, which were identical to the histological composition. The mean interspace between facets was set at 0.1 mm. The detailed material properties used for the components of the intact FE model were referenced in the previous literature (Table 1) (Tan et al., 2021a). Seven ligaments connecting the vertebrae were modeled as tension-only, three-dimensional spring elements (Table 2) (Naserkhaki et al., 2018). The validated procedure was the same as previously reported methods (Tan et al., 2021a).

**TABLE 1:** Element type and number of the components in the intact model.

Component	Element type	Young’s modulus (MPa)	Element number
Vertebra body			
Cortical bone	C3D8R	12,000	3,437
Cancellous bone	C3D4	100	214,269
Endplate	C3D8R	24	6,336
Posterior element	C3D4	3,500	366,252
Facet cartilage	C3D8RH	Neo-Hookean	6,495
Disc			
Annulus ground	C3D8RH	Mooney–Rivlin	6,000
Nucleus pulposus	C3D8RH	Mooney–Rivlin	7,200
Annulus fibers	Spring	Calibrated stress–strain curves	14,400

**TABLE 2:** Details of ligaments in the intact model (Naserkhaki et al., 2018).

Ligament	Element type	Element number	Origin and insertion	Length^a^
ALL	5 parallel springs	25	Connecting the anterior side of the endplate from T12 to L5 and with attachment to discs	15.58
PLL	6 parallel springs	20	Connecting the posterior side of the endplate from T12 to L5 and with attachment to discs	7.66
FCL	8 parallel springs (each side)	80	Encasing the facet joints (each side)	2.17
FL	9 parallel springs	45	Connecting the inferior and superior laminae of adjacent vertebra	15.89
ISL	6 parallel springs	30	Connecting the inferior and superior edges of adjacent spinous processes	8.85
SSL	1 parallel spring	5	Connecting the posterior tips of adjacent spinous processes	27.49
ITL	2 parallel springs (each side)	20	Connecting the inferior and superior edges of the adjacent transverse process (each side)	18.69

ALL, anterior longitudinal ligament; PLL, posterior longitudinal ligament; FCL, facet capsular ligament; LF, ligamentum flavum; ISL, interspinous ligament; SSL, supraspinous ligament; ITL, intertransverse ligament.

a The length of each ligament of a represented segment L4-L5.

### Generation of the Surgical Model

The primary surgical intervention was set as instrumented transforaminal lumbar interbody fusion (TLIF) at the level of L4-L5 with a traditional posterior dual-rod construct. Semifacetectomy was performed to excise the right superior and inferior facets of the L4-L5 level. Thereafter, a partial intervertebral disc was removed, and the prepared interspace between endplates was filled with a polyetheretherketone cage surrounded by an autograph bone graft. Subsequently, L3-L5 segments were fixed using bilateral transpedicular screws and rods.

The revision surgical intervention was set to address ASD located at the level of L2-L3. The decompression and fusion procedure was performed with TLIF. However, posterior instrumentation was different among the groups. All primary implants were retained in three constructs, and the differences are listed as follows:

DRCm: The extended fusion segments (L1-L2) were instrumented by bilateral connector rods attached to the ends of the primary rods and locked by set screws.

SRC: The L1-L2 level was instrumented with two bilateral conventional shorter rods that were connected to the primary rods by side-by-side connectors and lateral satellite rods.

**FIGURE 2:**
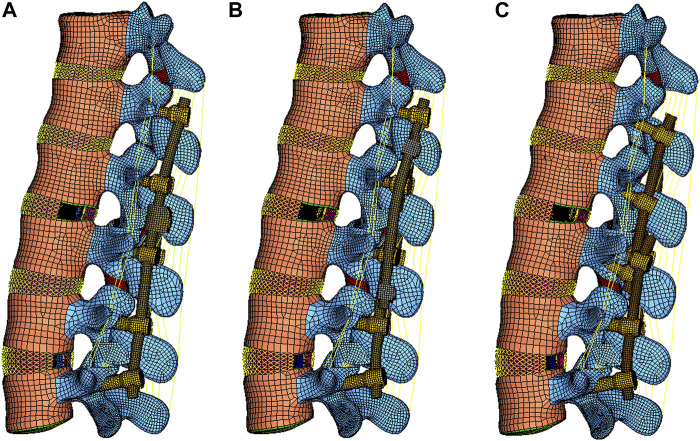
Lateral views of the RS FE model constructed using the DRCm **(A)**, SRC **(B)**, and CBTC **(C)**.

CBTC: The L1-L2 level was instrumented using the technique of the CBT. Screws implanted into the vertebral body of L1-L3 were placed *via* CBT according to the reported literature (Mullin et al., 2016). Briefly, the entry point of the CBT screws started at the lateral part of the pars interarticularis and followed a mediolateral, caudocephalad directed path. Thereafter, bilateral rods connected to the screws were implanted into L1-L3. A total of four screws were placed in L3 (Figure 2).

Implants applied in primary surgery and revision surgery, including screws, rods, connecters (Ti6Al4 V) and cages, were designed and constructed using SolidWorks (Dassault Systèmes, MA, United States). C3D8R was applied to mesh screws, rods, connectors, and cages. Thereafter, all components were imported into Abaqus 6.11 (Dassault Systèmes, MA, United States) for further analysis.

### Contact Definition

The interface between facet joints was defined as a frictionless surface-to-surface contact. The contact surface of the pedicle screw and rod, pedicle screw and vertebral body, and cage and endplate were all modeled as tie constraints according to the reported literature (Zhang et al., 2018; Tan et al., 2021a).

### Loading and Boundary Condition

During the loading process, the inferior surface of the endplate of the L5 vertebra was fully constrained in six directions. A pure moment of 7.5 Nm was applied to the node coupled with the superior surface of the endplate of the T12 vertebra. Then, a follower load of 500 N was applied to the revision FE model. The follower load is a physiological compressive load along the axis of the lumbar spine, in which intermediate nodes of each endplate are coupled to the endplate surface and connector elements built through these nodes. The follower load was applied to each segment through these connector elements.

### Data Analysis

The changing characteristics of maximum von Mises stress (VMS) on the annulus fibrosus and cages and ligament force of the interspinous ligament (ISL), supraspinous ligament (SSL), and ligamentum flavum (LF) were used to evaluate the spinal kinematic data under the loading direction of flexion (FL), extension (EX), left bending (LB), right bending (RB), left axial rotation (LAR), and right axial rotation (RAR) after the revision surgery was performed using the DRCm, SRC, and CBTC, respectively.

## Results

### Validation

The intact T12-L5 FE model was validated in our previous study by comparing the predicted range of motion (ROM) and disc compression with reported cadaveric studies. The predicted ROM under a loading pure moment without a preload and predicted disc compression under a 1200 N follower preload of the present FE model were within the range of reported data from cadaveric studies (Tan et al., 2021a).

### Maximum VMS on Annulus Fibrosus

**FIGURE 3:**
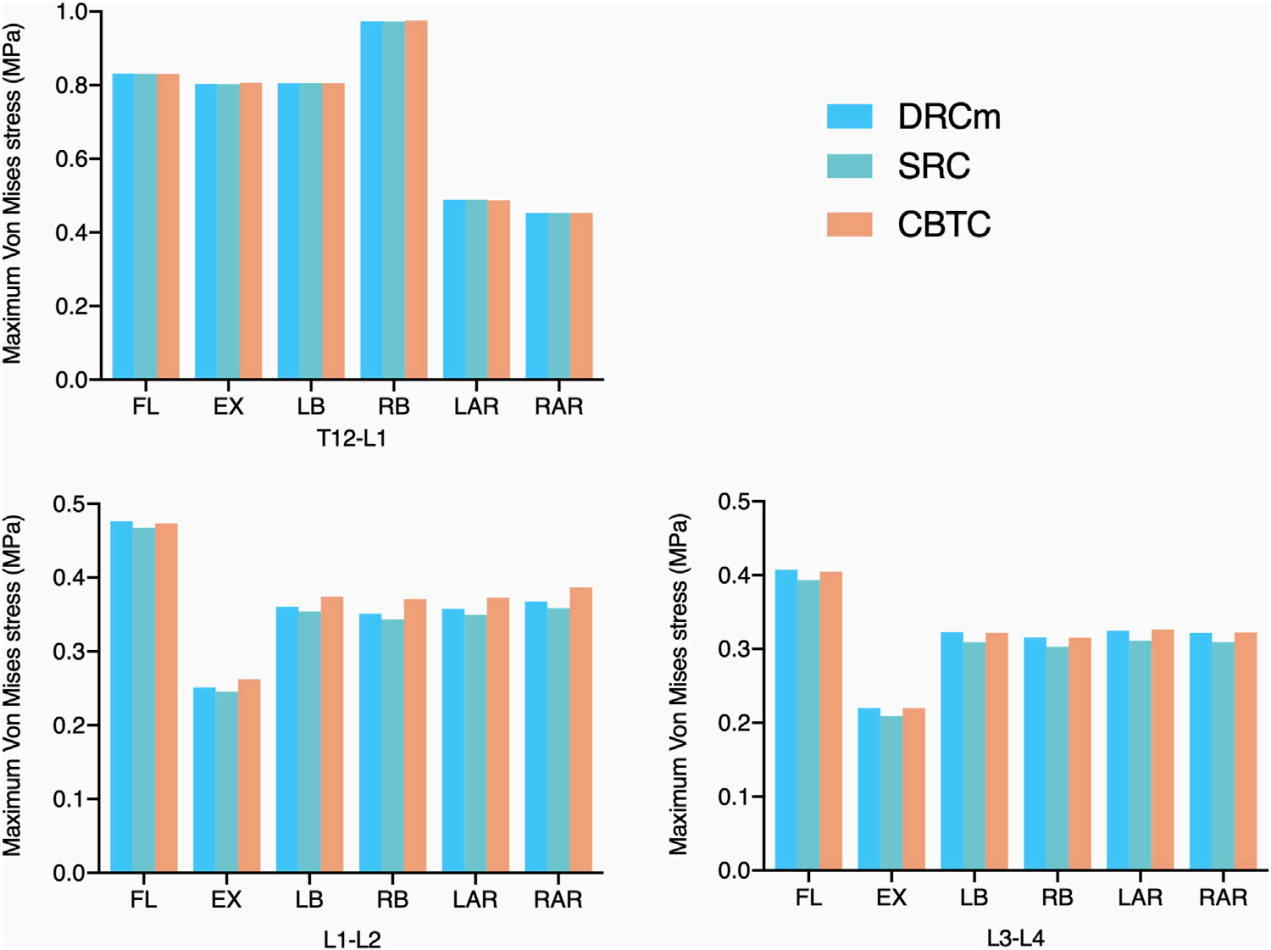
Maximum VMS was distributed on the annulus fibrosus after RS was instrumented using the DRCm, SRC, and CBTC in the loading direction of FL, EX, LB, LAR, and RAR.

As shown in Figure 3, the overall changing tendency of the maximum VMS on the annulus fibrosus was similar among the DRCm, SRC, and CBTC after RS in the six loading directions. In addition, the maximum VMS on the annulus fibrosus at T12-L1, L1-L2, and L3-L4 after RS was compared to the intact model. The maximum VMS of the three models after revision surgery on the annulus fibrosus at the level of T12-L1 was slightly changed, with an increase of 0.10–0.16% and 0.42–0.47% at FL and LB, respectively, and reductions of 0.44%–1.01%, 1.18%–1.53%, 0.49%–1.00%, and 1.20–1.31% at EX, RB, LAR, and RAR, respectively. A significant reduction in the maximum VMS on the annulus fibrosus of the three constructs occurred under the loading direction of extension, which was 66.86–69.00% and 64.34–66.06% for L1-L2 and L3-L4, respectively, compared to that of the intact model. At L1-L2, the degree of reduction in extension was 49.30%–53.07%, 43.90%–46.90%, and 21.10–22.03% under the loading directions of RB, LB, and FL, respectively. The degree of reduction under LAR and RAR was within 4%. At the level of L3-L4, the degree of reduction in extension was 52.74%–54.63%, 49.19%–51.36%, 30.25%–32.72%, 11.99%–16.22%, and 9.18–12.82% under RB, LB, FL, LAR, and RAR, respectively.

### Maximum VMS on Cage

**FIGURE 4:**
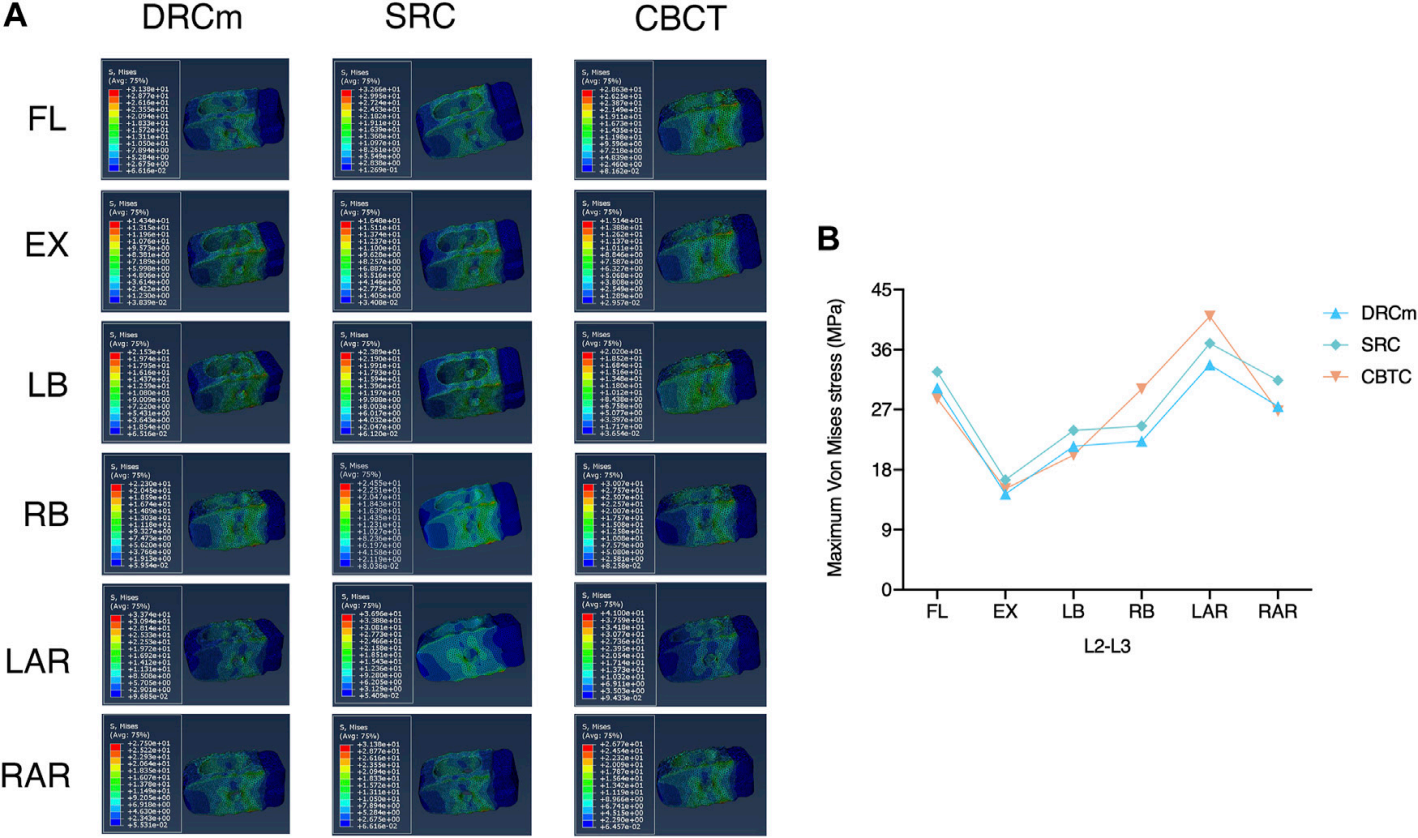
Distribution characteristics of the maximum VMS on the cage at the revision level after RS **(A)** and its tendency for change among the three constructs **(B)**.

**FIGURE 5:**
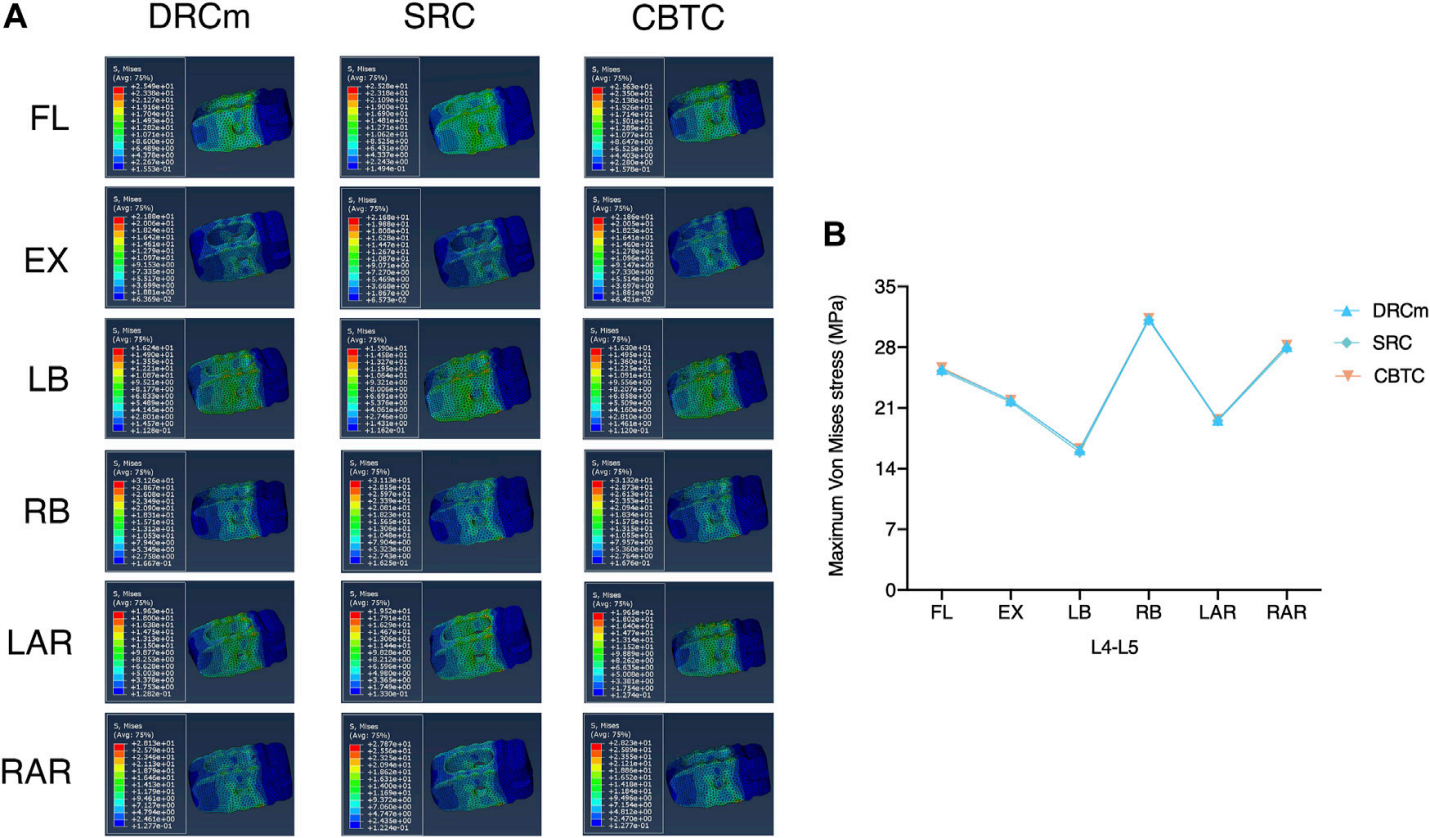
Distribution characteristics of the maximum VMS on the cage at the previous surgical level **(A)** and its tendency for change among the three constructs **(B)**.

The predicted VMS on the cages at the RS level (L2-L3) and PS level (L4-L5) are shown in Figures 4, 5. The maximum VMS was observed on the denture(s) of the cage, which were anchored to the bony endplate during surgical intervention under six loading directions in the three groups. The distribution profiles of VMS on the cage at the L4-L5 level were nearly identical among the DRCm, SRC, and CBTC (Figure 5A). In addition, the changing characteristics of the maximum VMS detected on the cage of PS at the L4-L5 segment after RS among the groups were almost the same (Figure 5B). However, at the revision level of L2-L3, a similar distribution profile of VMS was detected between the DRCm and SRC, while a slight distinction between the CBTC and the former two was observed, especially at the loading direction of RB and LAR (Figure 4A). Furthermore, changes in the maximum VMS between the DRCm and SRC were nearly equal, while that of the CBTC was distinguished with both of them in the loading direction of RB and LAR (Figure 4B).

### Ligament Force

**FIGURE 6:**
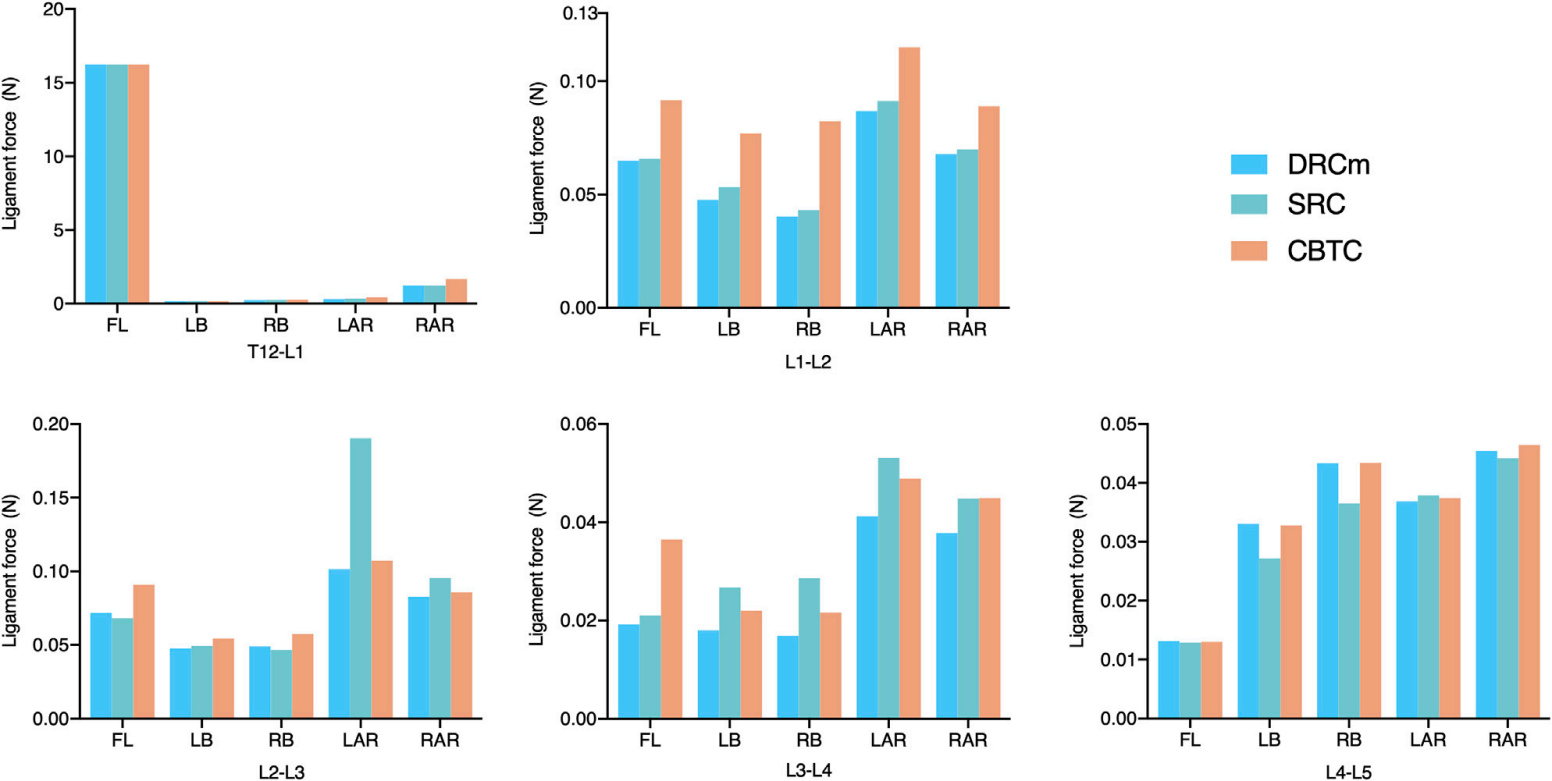
Ligament force of the ISL in the loading direction of FL, LB, RB, LAR, and RAR after RS instrumentation using the DRCm, SRC, and CBTC.

**FIGURE 7:**
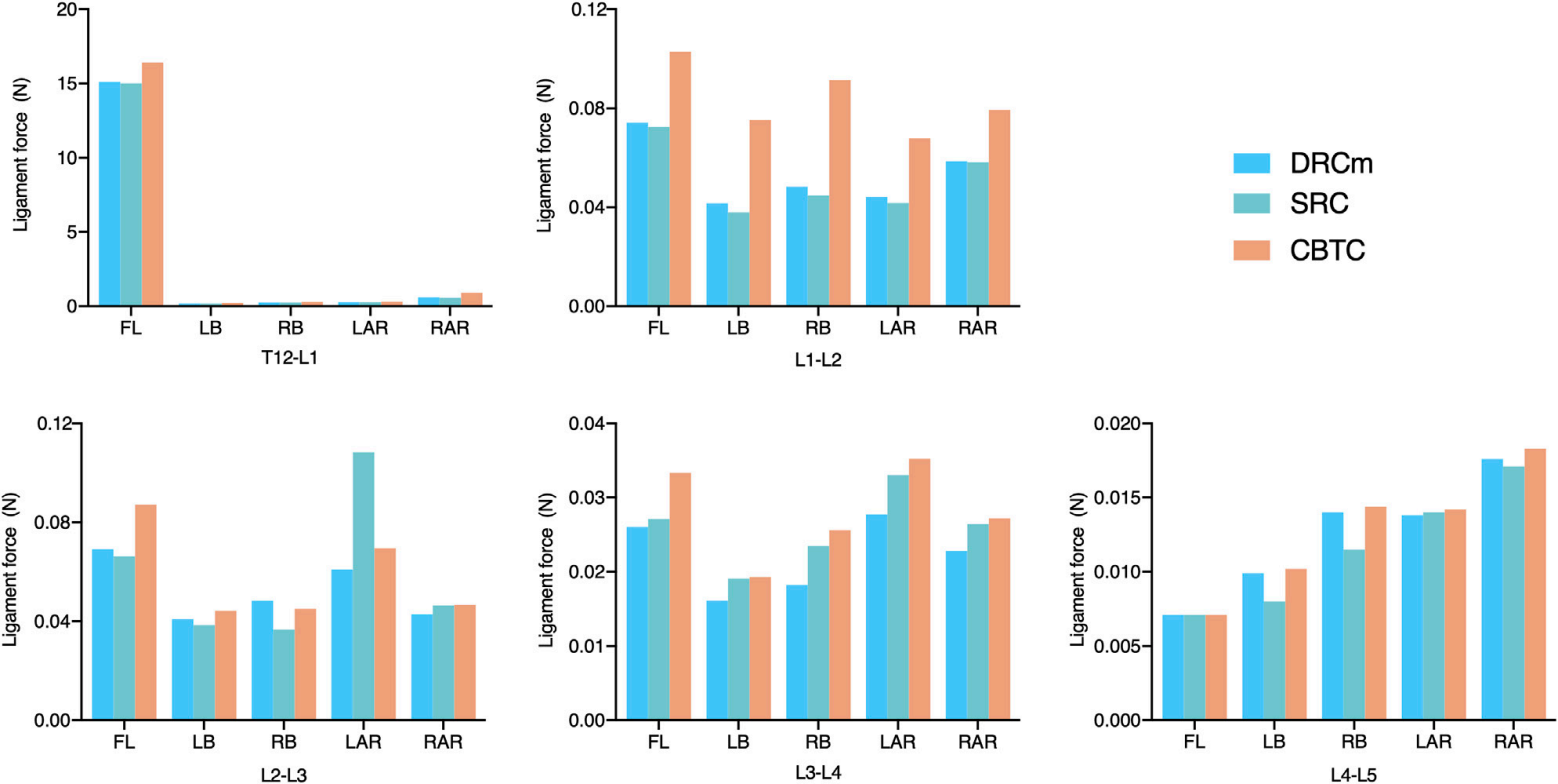
Ligament force of the SSL in the loading direction of FL, LB, RB, LAR, and RAR after RS instrumentation using the DRCm, SRC, and CBTC.

**FIGURE 8:**
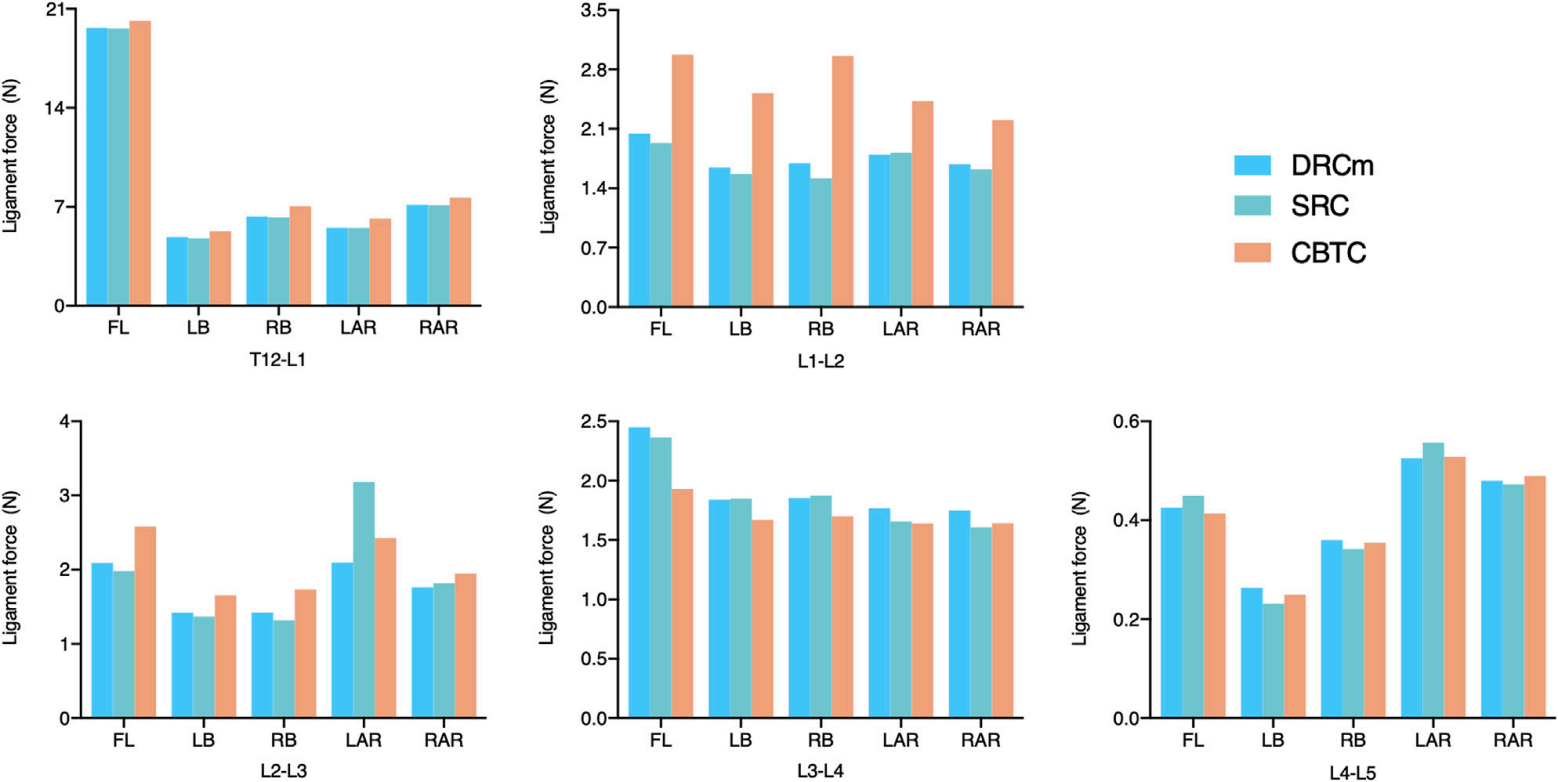
Ligament force of FL in the loading direction of FL, LB, RB, LAR, and RAR after RS instrumentation using the DRCm, SRC, and CBTC.

Ligament forces generated in the ISL, SSL, and LF under different loading directions were also compared among the three different constructs. The ligament forces of the ISL, SSL, and LF were markedly reduced when the movable spinal segments were fixated (unfixed T12-L1 vs*.* fixed segments; Figures 6–8). As shown in Figure 6, the ligament force in the ISL in the three different surgical constructs was reduced to an approximately equal value except for that of the CBTC at the level of L1-L2. Similar phenomena regarding the ligament force of the SSL and FL in the CBTC were also detected at L1-L2, while changes in the SSL and FL at other levels were approximately identical among the three constructs (Figures 7, 8). In addition, the reductions in the ISL (Figure 6), SSL (Figure 7), and LF (Figure 8) between the DRC and CRC were nearly the same. However, the ligament force of the ISL and SSL produced at L3-L4 in the SRC and CBTC was slightly larger than that in the DRCm.

## Discussion

Posterior spinal instrumentation is a commonly accepted intervention to restore spinal stability in individuals with spinal disorders (Kaiser et al., 2014). RS performed secondary to primary spinal fixation is a common surgical intervention to 1) address secondary diseases such as ASD and proximal or distal junctional kyphosis after deformity correction (Maruenda et al., 2016; Nicholls et al., 2017) and 2) rescue failed instrumentation due to rod fracture or nonunion (Yamato et al., 2018; Sherif and Arlet, 2020). Many constructs with different configurations such as the DRCm (Tan et al., 2021b), SRC (Buell et al., 2018; Jung et al., 2019), and CBTC (Rodriguez et al., 2014; Lee and Shin, 2018) have been applied in the aforementioned surgical interventions. One distinguishing application of the DRCm, SRC, and CBTC in RS is that the previous implants can be retained at their original site, which is different from that of the traditional surgical intervention in RS where the previous rods are removed. In such case, the primary surgical site has to be reincised to expose the previous rods, where soft tissues have already been disturbed. This traditional surgical intervention in RS is more complicated than the PS and correlated with an increase in surgical duration, blood loss, and possibility of complications (Zheng et al., 2002; Tan et al., 2021b).

In the present study, a validated FE model was constructed to detect the probable mechanical disparities among the DRCm, SRC, and CBTC using parameters including the VMS on the annulus fibrosus and cages and the ligament forces to further demonstrate the effect of different revision rod locations. Due to the complexity of the different clinical scenarios mentioned above, one relatively simple but common condition in clinical practice, ASD, was used to evaluate their biomechanics. ASD is more likely to develop in the segment(s) proximal to the previously fixed level (Lee et al., 2009). Therefore, RS for ASD is more frequently performed at the proximal segment(s) of the previous surgical segment. Thus, a previously validated intact T12-L5 FE model was applied and upgraded in the present study, in which the L3-L5 levels were instrumented to simulate the PS, and the L1-L2 level was instrumented to simulate the RS.

VMS is a parameter that has been used in many FE analyses to evaluate the effect of loading on the tissue. A higher risk of failure is associated with an increase in VMS (Li et al., 2017; He et al., 2021). In this study, VMS was used to index stress distributions on annulus fibrosus and cages as a reflection of the stabilization of the three configurations. In the uninstrumented T12-L1 segment, changes in VMS on the annulus fibrosus were nearly equal among the three models despite minor increases and decreases being detected when compared to those of the intact model. At the instrumented L1-L2 and L3-L4 segments, the VMS on the annulus fibrosus of the DRCm and CBTC under the six loading directions was almost the same, while that of the SRC was relatively less than that of the other two models in each loading direction. These results indicated that the biomechanics of these models were comparable to each other in RS. This significant reduction observed after the spine was instrumented was in accordance with that reported in another study, which might be due to the loading being dispersed through the instrumentation (Melnyk et al., 2012). The pronounced reductions detected under FL and EX might be due to the restriction of the backward and forward movements by spinal fixation.

The cage could support immediate axial loading after spinal decompression, and posterior instrumentation could reduce the stress distributed throughout the cage (Galbusera et al., 2012). Stiffer fixation could obviously reduce the stress transferred through the cage, while flexible instrumentation could generate more stress concentration on the cage (Galbusera et al., 2012; Fan et al., 2019). In the present study, the VMS that was placed on the cage at the facetectomy level (L2-L3) in the loading direction of RB and LAR in the CBTC was larger than that in the DRCm and SRC. Therefore, the CBTC might be a less rigid construct than the DRCm and SRC (Figure 4). This disparity in the VMS distribution might be attributed to the involvement of the resected facet by the loading directions of RB and LAR, while the CBTC instrumentation could not completely counteract the effect of the facetectomy. However, either the VMS distribution on the cage at the L4-L5 level (Figure 5A) or the detected value (Figure 5B) after RS was nearly the same in the three FE models. This might be because of instrumentation of the segments by conventional dual-rod constructs in the PS.

Ligaments play an important role in maintaining spinal stability (Widmer et al., 2020). Therefore, in many studies, the ligament force has been applied to assess spinal stability (Arshad et al., 2017; Naserkhaki et al., 2018; Buell et al., 2019). The SSL, ISL, and FL are parts of the posterior ligamentous complex that function as posterior tension bands to protect the spine from excessive movements in the flexion-distraction, rotation, and translation directions (Chen et al., 2017). In the present study, ligament forces of the SSL, ISL, and FL were adopted to predict spinal stability after instrumentation of the three constructs. As shown in Figures 6, 7, the overall changing tendency of the ligament force between the SSL and ISL was approximately identical at each segment. This might be due to their adjacent anatomical locations as the former is distributed superior to the spinous processes and the latter is distributed between the spinous processes. However, the ligament forces detected at segments L1-L2, L2-L3, and L3-L4, where the vertebrae were instrumented by the CBTC, were larger than those detected at the same level but instrumented by the DRCm and SRC. A similar changing tendency was detected in the FL, with a relatively higher ligament force detected at the L1-L2 and L3-L4 segments (Figure 8). The predicted results of the ligament force further indicated less rigidity fixation of the CBTC, which might retain a relatively greater motion of segments in RS.

Facetectomy is an intended facet joint removal and an indispensable procedure in TLIF to complete surgical intervention (Snyder et al., 2019). However, the facet joint plays an important role in protecting the spine from excessive movements of flexion, axial rotation, and forward displacement (O'Leary et al., 2018; Widmer et al., 2020). Therefore, facetectomy could inevitably alter spinal kinematics and biomechanics, and the impaired condition would be correlated with the grade of resection (O'Leary et al., 2018; Ahuja et al., 2020). It has been reported that destruction of the lumbar facet joints could transfer axial loads to the adjacent disc, which could conceivably accelerate the degeneration of the overburdened lumbar disc (O'Leary et al., 2018). Therefore, to restore spine stability after facetectomy, fusion under the assistance of instrumentation is needed (O'Leary et al., 2018). In the present study, the SRC, DRCm, and CBTC were investigated in RS. One difference of these constructs was that their revision rod was placed outside, alongside, and inside the previous rod at the facetectomy level, with a distance gradually approximating the sagittal plane of the facet joint. However, whether facetectomy affects the stability of constructs is unknown.

According to the predicted results of the VMS and ligament force, the DRCm, SRC, and CBTC can provide sufficient biomechanics to maintain spine stability after RS with previous implants retained. The mechanical property of the DRCm was nearly equal to that of the SRC, which indicated that the rod position located either alongside or outside the previous rod at the level of facetectomy might not affect the stability of the final spinal construct after RS. The CBTC was a less rigid instrument than the DRCm and SRC, which might be attributed to the different methods of connecting previously fixed segments (L3-L5) and RS (L1-L2) segments. In the DRCm and SRC, the two parts were connected with rigid metal components (rods and/or connecters) to form an integral fixation from L1 to L5. In the CBTC, the two parts were connected by additional CBT screws implanted into the L3 vertebra. However, the final spinal construct in the CBTC could also be recognized as two separate instrumentations from L1-L3 and L3-L5, although the L3 vertebra played a role similar to the titanium rod in the DRCm and SRC. Therefore, the movement axis of the CBTC might differ from that of the DRCm and SRC when applying pure movement plus a follower load on the T12 endplate under six loading directions while constraining L5. This might be attributed to the less rigid fixation of the CBTC compared to that of the DRCm and SRC. However, further study is needed to evaluate the biomechanics of the construct that binds L1-L3 and L3-L5 together.

The DRCm is a modified conventional dual-rod fixation that has been proven to be mechanically adequate for RS and has been applied in clinical practice (Tan et al., 2021a; Tan et al., 2021b). Clinical results indicated that the DRCm could shorten surgical duration, decrease blood loss, reduce RS-related complications, and generate favorable surgical outcomes compared to those of conventional dual-rod RS (Tan et al., 2021b). The SRC has been applied in clinical practice to deal with spinal deformity involving osteotomy and long segment instrumentation and the rescue of previous rod fracture (Buell et al., 2018; Zhu et al., 2018; Jung et al., 2019; El Dafrawy et al., 2020). The reported advantages of the SRC include dispersing the rod stress concentrated at the osteotomy site, enhancing the stability of constructs and reducing the occurrence of PJK (Hyun et al., 2014; Zhu et al., 2018; Gelb et al., 2021). However, extensive surgical site exposure and related complications, rod fracture at the rod-connecter site, and additional medical expenses are still concerns when using SRCs (Hyun et al., 2014; Jung et al., 2019). The CBT is a relatively new spinal fixation technique involving a unique caudocephalad and medial-to-lateral screw trajectory (Santoni et al., 2009). The biomechanical properties of CBT screws are superior to those of traditional pedicle screws (Santoni et al., 2009; Sansur et al., 2016; Zhang et al., 2019). Therefore, CBT is a better choice in treating osteoporotic patients (Ueno et al., 2013). In addition, due to the method of placing screws, CBT could be performed in a smaller operative corridor to avoid extensive surgical dissection but generate equal surgical outcomes to traditional pedicles with less invasiveness, shorter surgical time, and lower incidence (Sakaura et al., 2018; Sakaura et al., 2019). However, skilled screw placement techniques, clear vertebral landmarks, and image navigation might be necessary to achieve safe and satisfactory CBT screw placement (Rodriguez et al., 2014; Matsukawa et al., 2015; Tan et al., 2019). The findings of the present study revealed that all of these constructs could provide good stability in RS. However, the choice of the construct to be applied depends on the condition of the patients, surgeons, and facilities; the advantages and disadvantages of each construct are discussed above. For instance, the DCRm might be a choice for an older patient who cannot endure extensive surgical exposure (a shortcoming of the SRC), or the CBTC technique may not be available.

The limitations of the present study are as follows: first, although FE analysis is a good method to assess the biomechanics of spinal constructs, simplification is a common shortcoming of spinal FE analysis. The simplified FE model in the present study might not simulate the effect of the actual psychological situation on spinal constructs. In addition, the FE model was constructed based on the CT data of a 30-year-old healthy male subject, which might not fully simulate the condition of an ASD patient. Therefore, further investigation is needed. Second, clinical scenarios of applying different constructs were simplified to ASD due to their varieties and complexities, and further studies investigating applications of the mentioned constructs in more complicated situations are needed to further assess their biomechanics. Third, the effect of revision rods placed inside, alongside, and outside the previous rod was compared among configurations that have been applied in clinical practice rather than in one configuration with different rod positions, which has recently been unavailable in clinical practice; thus, further study needs to be performed. Fourth, the ligament force was used as an evaluating parameter when a precondition of the related ligament was integrated into the present study; however, the ISL and SSL might be destroyed and even resected at the decompressed lumbar level if the spinous process was intended to be removed. Therefore, actual surgical scenarios may not have been precisely represented in this study.

## Conclusion

The revision rod position could not affect the mechanical properties of the DRCm, SRC, and CBTC. All of these constructs were sufficient to provide safe and satisfactory fixation in RS without disturbing the prior implants. Although the CBTC was less rigid than the DRCm and SRC in providing spinal stabilization, it was not correlated with the position of the revision rods but was associated with the type of construction of this configuration. The surgical procedure using the DRCm in RS is more convenient than using the SRC and CBTC. The clinical application of these configurations depended on full consideration of their technique requirements and actual clinical and surgical conditions.

## Data Availability

The raw data supporting the conclusion of this article will be made available by the authors, without undue reservation.
